# Impact of *Saccharomyces cerevisiae* DSM 34246 (Canobios-BL) var. *boulardii* Supplementation on Nutritional Status and Fecal Parameters in Healthy Breeding Adult Cats

**DOI:** 10.3390/vetsci12010044

**Published:** 2025-01-10

**Authors:** Nicolò Lonigro, Elisa Martello, Natascia Bruni, Mauro Bigliati, Annalisa Costale, Ilaria Lippi, Giorgia Meineri, Francesca Perondi

**Affiliations:** 1 Department of Drug Science and Technology, University of Turin, 10124 Turin, Italy; nicolo.lonigro@unito.it (N.L.); annalisa.costale@unito.it (A.C.); 2 Division of Epidemiology and Public Health, School of Medicine, University of Nottingham, Nottingham NG5 1PB, UK; 3 Candioli Pharma S.r.l., 10092 Turin, Italy; natascia.bruni@candioli.it (N.B.); mauro.bigliati@candioli.it (M.B.); 4 Department of Veterinary Science, University of Pisa, 56122 Pisa, Italy; ilaria.lippi@unipi.it (I.L.); f.perondi87@gmail.com (F.P.); 5 Department of Veterinary Sciences, School of Agriculture and Veterinary Medicine, University of Turin, 10095 Turin, Italy; giorgia.meineri@unito.it

**Keywords:** pet, probiotic, feline, natural products, gut health

## Abstract

The feed additive *Saccharomyces cerevisiae* DSM 34246 (Canobios-BL) var. *boulardii* was tested on adult cats (breed: Chartreux) in order to show its effect on the intestinal health. A total of 14 healthy cats (4 males, 10 females) were included. The animals were randomly assigned to a control group, group CTR, and a treated group, group SACC. Both groups were fed with a dry commercial diet two times a day, and the feed additive was added (at a concentration of 5 × 10^9^ CFU/kg of food) to the diet for group SACC and a placebo (Maltodestrin) for group CTR. This randomized blinded controlled trial lasted 35 days and was divided into six experimental time points. Body weight (BW), body condition score (BCS), muscle condition score (MCS), fecal score (FS), fecal score measured with a penetrometer (FSp), fecal dry matter (DM), fecal humidity (UM), and fecal IgA (IgA) were measured using a specific method of analysis. Water intake (WI) was monitored weekly. Analysis of variance, Kruskal–Wallis test, and pairwise two-sample Wilcoxon test were used for the statistical analysis according to the type of data. The results showed significant differences between the two groups for some of the tested parameters: BCS, FS, FSp, DM, UM, and IgA. This suggests the positive effect of supplementation with *S. boulardii* on gut health and on the maintenance of good physiological and biological conditions in healthy cats living in a breeding environment.

## Simple Summary

This study tested *Saccharomyces cerevisiae* DSM 34246 (Canobios-BL) var. *boulardii* on adult Chartreux cats to evaluate its impact on gut health. Fourteen healthy cats (4 males, 10 females) were randomly assigned to a control (CTR) and treated (SACC) group. Both were fed a dry diet twice daily, with the additive given to the SACC group (5 × 10⁹ CFU/kg) and a placebo to the CTR. Over 35 days, body weight, condition scores, fecal parameters, and water intake were measured. Statistical analysis showed significant improvements in body condition, fecal parameters, and IgA, indicating that *S. boulardii* positively affected gut health in these cats.

## 1. Introduction

Recent studies in veterinary science highlight the close relationship between pet health and gastrointestinal health [1]. The gut microbiota plays diverse roles, including digestion and nutrient absorption, defense against pathogens, and the development of a healthy intestinal epithelium and immune system [2,3]. Probiotics are defined as “live microorganisms” that confer positive effects on the host’s health and mitigate dysbiosis due to stress stimulation [1,3,4,5]. They are largely used to maintain gastrointestinal health, and the most commonly studied are Lactobacillus, Bifidobacterium, and yeasts [1,4,6]. Yeast products, in particular, may also exhibit probiotic properties [4]. The baker’s yeast Saccharomyces cerevisiae does not appear to offer significant advantages; however, *S. cerevisiae* var. *boulardii* has been shown to be effective in treating acute and chronic gastrointestinal diseases in humans [4,7,8]. *S. boulardii* is the only yeast used as a probiotic, with its properties well supported by scientific evidence in humans [4]. Recently, yeast products, particularly *S. boulardii*, have also been used as probiotics in pets [3,5,9,10]. *S. boulardii* has demonstrated clinical effectiveness in dogs with chronic gastrointestinal diseases [10] and has positively impacted the nutritional and stress status of healthy dogs [3]. The mechanisms through which yeast probiotics support gastrointestinal function are not yet fully understood, but evidence supports the fact that they improve intestinal barrier function, aid in the regeneration of intestinal tissue, inhibit the colonization of pathogenic microorganisms, and regulate immunity [3,5]. However, studies on the application of *S. boulardii* in cats are very limited [1,5]. Li and colleagues demonstrated that using this probiotic in healthy cats can promote gut health by modulating gut microbes, improving microbiota, reducing inflammatory conditions, and enhancing antioxidant status [1]. Conversely, Zhang and colleagues did not show any improvement in kittens fed with *S. boulardii* compared to those receiving dietary supplementation with enzymolysis seaweed powder [5].

Given the limited knowledge of the efficacy of *S. boulardii* on the gut health of cats, the objective of this study is to explore the efficacy and safety of the feed additive *S. cerevisiae* DSM 34246 (Canobios-BL) var. *boulardii*, classified under the functional group “gut flora stabilizers” by EFSA [11], on the fecal parameters of healthy cats.

## 2. Materials and Methods

### 2.1. Animals and Study Design

The study is a randomized double-blinded controlled trial. We included a group of 14 adult Chartreux cats, aged between 3 and 6 years. In detail, 10 cats were female and four were male with a mean weight of 4 kg. Throughout the study period of 35 days, the health of all cats was regularly assessed by the same veterinarian, and all cats maintained good health. The cats were managed according to standard kennel procedures, with each cat housed in a room (one cat per room). The kennel provided both indoor and outdoor areas, each measuring 6 square meters. Breeding activities took place in a secluded open-air area to minimize exposure to external stressors, such as roads and urban noise. Routine cleaning and disinfection procedures were conducted according to established breeding standards. Daily observations of the cats’ health were carried out by the breeder, with weekly assessments performed by the veterinarian. Cats were fed a dry commercial diet with no probiotics included (Royal Canin Sensible, humidity 4%, raw protein 32.5%, crude fat 23.5%, crude fiber 1.3%, crude ash 7.4%) in kibbles. Feed was given two times a day (morning and afternoon, 70 g food/cat/day) and water was available ad libitum [12], with each cat having a graduated water dispenser (Drinky Cat Beverino—Ferplast). The daily water intake (WI) of each cat was registered and evaluated in order to exclude fecal humidity (UM) variability due to the quantity of drinking water per day. All cats were randomly assigned to a control group (CTR; 2 males and 5 females) fed with complete and rationed dry commercial diet for 35 days, and a treated group (SACC; 2 males and 5 females), receiving the same diet, but supplemented with the feed additive *S. cerevisiae* DSM 34246 (Canobios-BL) var. *boulardii* in the form of powder [11]. Both the cats’ owner and the veterinarian assessing the animals and the analyst were not aware of the group belonging to the tested product or placebo. The intended concentration of *S. boulardii* as the active substance in the food was 5 × 10^9^ CFU/kg (250 mg/kg of additive). The experiment consisted of a 7-day adaptation period, followed by a 35-day data collection period divided into 5 time points each 7 days apart (from T0 to T5). Following the AAHA Nutritional Assessment Guidelines for Dogs and Cats [13], the parameters considered as indicators of the cats’ health status and considered in this study were body weight (BW), body condition score (BCS), muscle condition score (MCS), fecal score (FS), fecal score measured with a penetrometer (FSp), fecal dry matter (DM), fecal humidity (UM), and fecal immunoglobulin A (IgA). In case of any change in health status, pregnancy, pharmacological treatments, diet change, pathological or stress symptoms, and/or death, the patients would be excluded.

During the trial, animal welfare principles were carefully considered to minimize social stress while respecting the cats’ need to socialize with conspecifics and humans. Additionally, efforts were made to provide a suitably stimulating environment for the cats.

The health conditions of cats were examined weekly by the veterinarian from the beginning of the study. The breeder was informed of the design of the study and signed a written informed consent form. The experimental procedures used in this trial were reviewed and approved by the Bioethics Committee of the University of Turin (approval 156895, 14 April 2020).

### 2.2. Method of Data Collection

The evaluation of the nutritional status was performed by the same veterinarian following standard guidelines [13]. BW and BCS were recorded at t0 (day 0), t1 (day 7), t2 (day 14), t3 (day 21), t4 (day 28), and t5 (day 35). BCS is an effective assessment of body fat [14]; scores between 1 and 9 were assigned by visual examination and palpation of the animal at t0 and t5. A score of 4 or 5 represents the ideal score [14]. MCS [14] differs from BCS in that it evaluates muscle mass (graded as normal, mild loss, moderate loss, or severe loss). MCS assessment was performed at each experimental time and included visual examination and palpation of the temporal bones, shoulder blades, lumbar vertebrae, and pelvic bones. Evaluating muscle condition is important, as it negatively affects strength, immune function, and wound healing. Five fecal parameters—FS, FSp, DM, UM, and IgA—were evaluated for each time point (from T0 to T5).

In particular, FS was determined on fresh feces using a 7-point scale, just before the collection [15,16]. The measurement of fecal hardness, in kg/cm2, was performed on fresh stool with a Penetrometer 53220 FTA (GUSS Manufacturing, PTY Ltd., Cape Town, South Africa) following a technique already described by Davies and colleagues [15]. The amount of stool used for each analysis was at least 40–50 g, depending on the type and shape of stool itself. Fecal hardness was defined as the mean of three measurements. Fecal hardness was converted using a validated scale to obtain the FSp.

For the measurement of the UM (mean of two measurements), a 5–10 g stool sample was weighed and dried in an oven at a temperature of 105–110 °C for 20–24 h, then cooled into a desiccator for 20–24 h, and the dry sample was weighed. The result was expressed as a percentage.

For the measurement of DM, it was calculated from the UM through the following method: DM = 100 − UM. The result was expressed as a percentage.

The fecal IgA level was measured using a specific ELISA kit (enzyme-linked immunosorbent) (Cat IgA ELISA Quantitation Set, Bethyl Laboratories Inc., Montgomery, TX, USA). Aliquots of 1.3 g of stool (wet weight) were diluted 1:5 in fecal extraction buffer (20 mM CH_3_CO_2_Na and 3 mM CaCl_2_ [pH, 7.6]) containing a cocktail of proteinase inhibitors (tablets complete with proteinase inhibitors without EDTA, Roche Diagnostics GmbH, Mannheim, Germany; 1 tablet/25 mL). After homogenization by vigorous shaking for 30 min at approximately 23 °C, the suspensions were centrifuged for 20 min at 2100× *g* at 5 °C. The supernatants were collected using serum filters (Fisherbrand Serum Filter System (Model IB), Fisher Scientific Inc., Pittsburgh, PA, USA) and centrifuged for 30 min at 10,600× *g* at 23 °C. The final supernatants (fecal extracts) were stored frozen at –20 °C until analysis.

### 2.3. Statistical Analysis Methods

The statistical analysis conducted in the study varied depending on the type of parameter (e.g., categorical or numerical). The parameters BW, FSp, IgA, DM, and UM were analyzed using analysis of variance (ANOVA) based on a repeated measures model implemented through the mixed procedure (PROC MIXED MODEL SAS 9.4, 2013).

The statistical model was structured as follows:

y = μ + Si + Gj + Tk + GTjk + ejkn,

where

- y = dependent variable;
- μ = overall mean;
- Si = fixed effect of the sex (i = F; M);
- G = fixed effect of the treatment (j = 0, 1);
- Tk = fixed effect of the kth time (k = 1, 6).
- GTjk = fixed effect of the interaction between the jth treatment and kth time;
- ejkn = error.

Time was treated as a repeated measurement and replicated with the groups as repeated subjects. The autoregressive covariance method was used for the covariance structure. Least-square means were separated using Student’s *t*-test. The BCS and FS of the CTR and SACC groups were compared using the Kruskal–Wallis test (UCLA) for the overall experimental period using PROC NPAR1WAY (SAS 9.4). If significant results were found, multiple comparison analysis based on pairwise two-sample Wilcoxon comparisons was conducted. Test statistics from two-tailed tests with *p*-values < 0.10 were considered significant.

## 3. Results

All the cats remained healthy throughout the study, and no cases of mortality were recorded. None of the animals involved in the study received any pharmacological treatment during the 15 days prior to the study’s commencement.

The MCS was defined as normal for all cats at each time point throughout the study period in both the SACC and CTR groups; consequently, no statistical analysis was performed.

The effects of adding the supplement to the diet on BW and BCS are presented in Table 1.

The BW of the cats did not show statistically significant differences between the groups at any time point.

However, the BCS significantly decreased in the SACC group (but not in the CTR group) over time.

Table 2 shows the results for FS, FSp, DM, UM, and IgA for both cat groups.

The FS was different between the groups only at t2, t3, t4, and t5. The FS in the SACC group reported a statistically significant decrease during the treatment period. On the other hand, the CTR group did not report a significant decrease in these values over time.

The FSp was different between the groups only at t2, t3, t4, and t5. The FSp of the SACC group significantly decreased from t0 to t5.

The DM measured for the SACC group showed a statistically significant increase during the treatment period. The CTR group did not report a significant increase in the values measured. The DM was significantly different between the groups only at t2, t3, t4, and t5.

The UM showed a statistically significant decrease in the SACC group during the study. On the other hand, the CTR group did not report a significant decrease. The UM was significantly different between the groups only at t2, t3, t4 and t5.

The IgA in the SACC group showed a significant increase over time during the study. The CTR group did not report a significant increase. The IgA measured reported significant differences between the groups only at t5.

The WI in the two groups during the study did not show a statistically significant variation. Both groups were similar at each time point, as summarized in Table 3.

Additionally, there were no changes in feed administration or consumption (feed intake—FI, Table 4).

## 4. Discussion

Yeast products such as probiotics have not yet been well studied in relation to cats [1,4,5]. In this study, we investigated the efficacy of a feed additive containing *S. boulardii* on the fecal quality parameters and nutritional parameters of healthy Chartreux cats.

At the beginning of the experiment, all cats involved in our study were healthy, and the administration of *S. boulardii* did not cause any short-term adverse effects, consistent with findings on the use of the same probiotic in pets reported by other authors [3]. The present results on cats confirm the product’s tolerability and safety and suggest its positive effect in enhancing fecal consistency and in reducing inflammatory conditions.

Specifically, the decrease in BCS in the subjects under treatment reflects a good maintenance of the nutritional condition, as also reported in previous research on dogs [9,17]. Some studies in dogs have shown that yeast products do not affect feed intake or BW but do result in an increased BCS [9,18].

Our results also demonstrated a positive effect on all fecal parameters tested (FS, FSp, UM, and DM), highlighting the effectiveness of *S. boulardii* supplementation in improving fecal consistency by reducing water content in the feces (Table 2), thus indirectly confirming positive effects on gut functionality.

In dogs, it has been proven that supplementing with yeast products results in higher fecal scores and improved fecal consistency (DM) compared to control groups [9]. For cats, the available data are limited, but one study showed that yeast products improved DM and digestibility [19].

*S. boulardii*, as a strain of *S. cerevisiae*, has strong probiotic properties and anti-pathogenic abilities [1]. Studies have shown that *S. boulardii* plays a significant role in preventing antibiotic-associated diarrhea [1] and in dogs with gastrointestinal diseases [10]. Two studies in cats have confirmed the positive effects of yeast products (*S. cerevisiae*) on feline gut health by modulating gut microbes and improving microbiota [1,5]. Dysbiosis can significantly impact intestinal health [20], and gut microbes are crucial for maintaining health and regulating the occurrence of various diseases in pets [1]. In fact, a significant increase in fecal IgA was observed in the group fed with *S. boulardii* after 35 days of treatment. Immunoglobulin levels can indirectly reflect an animal’s ability to resist exogenous pathogens and are critical parameters for evaluating immunity [5]. IgA is crucial for the local anti-infection action of the body’s mucosa [5]. Recently, fecal IgA has been suggested as a non-invasive marker of canine intestinal health [3,9,21].

In addition, veterinary studies on different animal species (dogs, cats, poultry) have shown that dietary supplementation with *S. boulardii* increases IgA levels in saliva, feces, and serum [5,9,22]. However, studies specifically regarding cats on the effects of *S. cerevisiae* on the immune function are very limited [9]. Matheus et al. [23] and Lima et al. [24] did not report any differences in cats supplemented with yeast products (*S. cerevisiae*) when comparing some hematological and inflammatory parameters involved in the immune response. On the other hand, studies in dogs confirm that supplementation with yeast products (*S. cerevisiae*) increases ileal and fecal IgA, indicating enhanced mucosal immunity and immunomodulatory properties [4,9,25]. These results in dogs are aligned with our findings on cats, demonstrating that direct dietary supplementation with *S. boulardii* may play a crucial role in anti-inflammatory activity and improve intestinal immune regulation in cats. Our study was limited by the small sample size and the inclusion of only one cat breed. Future research should include a larger, more diverse population of cats from various breeds and health conditions to strengthen the findings. Additionally, exploring other fecal and blood markers of immune function and inflammation (i.e., fecal calprotectin, platelets and white cells, cell phagocytic capacity, secretion of pro- and anti-inflammatory cytokines, acute phase proteins) [3,9] and examining the microbiota could provide valuable insights into animal gut health.

## 5. Conclusions

The supplementation of *S. boulardii* at the recommended dietary dosage of 5.0 × 10^9^ CFU/kg of feed used in adult healthy cats for 35 consecutive days significantly affected several parameters, including FS, FSp, DM, UM, BCS, and IgA. However, the supplementation did not cause a significant variation in BW in the animals. Based on the results obtained, *S. boulardii* is a promising supplement for cat diet for stabilizing the gut flora. Importantly, further exploration of the role of yeast products as probiotics in cats with intestinal diseases is necessary to improve feline intestinal health.

## Figures and Tables

**Table 1:** Body weight (BW) and body condition score (BCS) parameters measured in treated (SACC) versus untreated (CTR) cats at different time points during the study.

	t0	t1	t2	t3	t4	t5	Average	*p*-Value
**BW (Kg ± SE)**								
CTR	3.9 ± 0.1	4.0 ± 0.1	4.0 ± 0.1	4.0 ± 0.1	4.0 ± 0.2	4.1 ± 0.2	4.0 ± 0.1	1.00
SACC	4.1 ± 0.1	4.1 ± 0.1	4.1 ± 0.1	4.1 ± 0.1	4.2 ± 0.1	4.2 ± 0.1	4.1 ± 0.1	1.00
**BCS (score ± SE)**								
CTR	5.9 ± 0.2	5.5 ± 0.3	5.9 ± 0.2	5.6 ± 0.3	5.7 ± 0.4	5.6 ± 0.3	5.7 ± 0.1	0.86
SACC	5.8 ± 0.3	5.3 ± 0.4	4.7 ± 0.2	4.4 ± 0.1	4.2 ± 0.1	**4.1 ± 0.1**	4.7 ± 0.1	<0.0001

**Table 2:** Fecal score (FS), fecal score measured with a penetrometer (FSp), fecal dry matter (DM), fecal humidity (UM), and fecal IgA (IgA) parameters measured in treated (SACC) versus untreated (CTR) cats at different time points during the study.

	t0	t1	t2	t3	t4	t5	Average	*p*-Value
**FS (score ± SE)**								
CTR	4.4 ± 0.5	4.1 ± 0.1	4.1 ± 0.1	4.3 ± 0.2	4.3 ± 0.2	4.6 ± 0.3	4.3 ± 0.1	0.85
SACC	4.3 ± 0.4	4.3 ± 0.4	**3.1 ± 0.1**	**3.3 ± 0.2**	**3.1 ± 0.1**	**3.0 ± 0.0**	3.5 ± 0.1	0.0006
**FSp (score ± SE)**								
CTR	4.5 ± 0.5	4.4 ± 0.2	4.5 ± 0.2	4.4 ± 0.3	4.4 ± 0.2	4.6 ± 0.3	4.5 ± 0.1	1.00
SACC	4.4 ± 0.4	4.3 ± 0.4	**3.2 ± 0.2**	**3.1 ± 0.2**	**3.0 ± 0.1**	**2.7 ± 0.1**	3.4 ± 0.1	0.0001
**DM (% ± SE)**								
CTR	31.0 ± 5.7	33.0 ± 1.9	31.6 ± 1.8	34.4 ± 2.6	36.5 ± 3.4	32.7 ± 3.9	33.2 ± 1.4	0.89
SACC	32.4 ± 4.3	33.6 ± 3.7	**51.9 ± 3.9**	**49.9 ± 1.5**	**52.1 ± 2.2**	**58.0 ± 2.8**	46.3 ± 2.0	<0.0001
**UM (% ± SE)**								
CTR	68.6 ± 5.7	66.0 ± 1.8	67.2 ± 2.0	64.5 ± 2.4	62.7 ± 3.3	66.1 ± 3.8	65.8 ± 1.4	0.87
SACC	67.3 ± 4.4	65.6 ± 3.7	**46.9 ± 3.8**	**48.8 ± 1.5**	**47.1 ± 2.1**	**41.2 ± 2.9**	52.9 ± 2.0	<0.0001
**IgA (mg/g ± SE)**								
CTR	5.4 ± 0.3	5.9 ± 0.2	5.8 ± 0.2	5.6 ± 0.2	5.8 ± 0.2	5.5 ± 0.1	5.7 ± 0.1	0.64
SACC	5.8 ± 0.2	5.5 ± 0.2	5.6 ± 0.2	5.9 ± 0.2	6.1 ± 0.2	**6.6 ± 0.2**	5.9 ± 0.1	0.0084

**Table 3:** Water intake (WI) in liters in the treated (SACC) and untreated group (CTR) of cats.

Group	t0	t1	t2	t3	t4	t5	Average	*p*-Value
CTR	0.20 ± 0.01	0.18 ±0.01	0.20 ± 0.01	0.19 ± 0.01	0.20 ± 0.01	0.20 ± 0.01	0.20 ± 0.00	0.46
SACC	0.19 ± 0.01	0.18 ± 0.01	0.20 ± 0.01	0.20 ± 0.01	0.20 ± 0.01	0.21 ± 0.01	0.20 ± 0.00	0.60

**Table 4:** Feed intake (FI) parameters measured in treated (SACC) versus untreated (CTR) cats at different time points during the study.

	t0	t1	t2	t3	t4	t5	Average	*p*-Value
FI (g ± SE)								
CTR	-	330.0 ± 1.0	329.4 ± 0.8	329.2 ± 0.8	328.9 ± 0.6	329.4 ± 1.0	329.4 ± 0.4	0.92
SACC	-	329.6 ± 0.8	329.8 ± 0.8	328.6 ± 0.6	330.3 ± 0.6	329.2 ± 1.0	329.5 ± 0.4	0.60

## Data Availability

The data that support the findings of this study are available on request from the corresponding author (E.M.).
